# Natural Products Targeting PAD4 in NETosis: Structural and Mechanistic Insights into Direct and Indirect Inhibition

**DOI:** 10.3390/biom16030420

**Published:** 2026-03-12

**Authors:** Dong Oh Moon

**Affiliations:** Department of Biology Education, Daegu University, 201, Daegudae-ro, Gyeongsan 38453, Gyeongsangbuk-do, Republic of Korea; domoon@daegu.ac.kr; Tel.: +82-53-852-6992

**Keywords:** PAD4, NETosis, natural products

## Abstract

Peptidyl arginine deiminase 4 (PAD4) is a Ca^2+^-dependent enzyme that catalyzes histone citrullination and plays a central role in chromatin decondensation during neutrophil extracellular trap (NET) formation. Dysregulated PAD4-mediated NETosis contributes to the pathogenesis of diverse inflammatory and immune-related diseases, including autoimmune disorders, cancer, and thrombosis. Although several synthetic PAD4 inhibitors have been developed, their therapeutic application has been limited by issues related to selectivity, irreversible covalent reactivity, and suboptimal pharmacokinetic properties, prompting growing interest in natural products as alternative modulators of PAD4 activity and NETosis. This article presents a structural and mechanistic overview of natural products that target PAD4 and regulate NETosis. Based on enzyme kinetics, structural analyses, and functional validation, natural PAD4 modulators are classified into four categories: (i) active-site-directed inhibitors that bind within the U-shaped substrate tunnel, (ii) mixed and active-site-adjacent inhibitors that engage surface pockets flanking the catalytic site, (iii) allosteric and hybrid modulators that bind to regulatory regions distinct from the active site, and (iv) functionally validated PAD4 binders supported by biophysical and cellular evidence. Integration of structural, biochemical, and cellular data highlights that indirect or noncanonical modes of PAD4 regulation represent biologically coherent strategies for controlling pathological NETosis.

## 1. Introduction

PAD4 (encoded by the PADI4 gene) is a calcium-dependent enzyme that catalyzes the post-translational conversion of arginine residues into citrulline, a process known as citrullination or deimination [1,2,3]. Among the PAD family members (PAD1, PAD2, PAD3, PAD4, and PAD6), PAD4 is unique in that it contains a nuclear localization signal, allowing its translocation into the nucleus and direct modification of histones [4]. PAD4-mediated histone citrullination reduces the positive charge of arginine residues, resulting in chromatin decondensation and consequent alterations in chromatin structure and gene regulation [5,6].

One of the most well-characterized biological functions of PAD4 is its essential role in NET formation, a process known as NETosis [7,8]. During NETosis, PAD4-catalyzed citrullination of histones H3 and H4 promotes extensive chromatin decondensation, thereby facilitating the release of DNA fibers decorated with granular and cytoplasmic proteins into the extracellular space [7,9,10]. Consistently, genetic deletion or pharmacological inhibition of PAD4 markedly impairs NET formation, demonstrating that PAD4 activity is a critical determinant of efficient NETosis in neutrophils [8,11].

**Figure 1:**
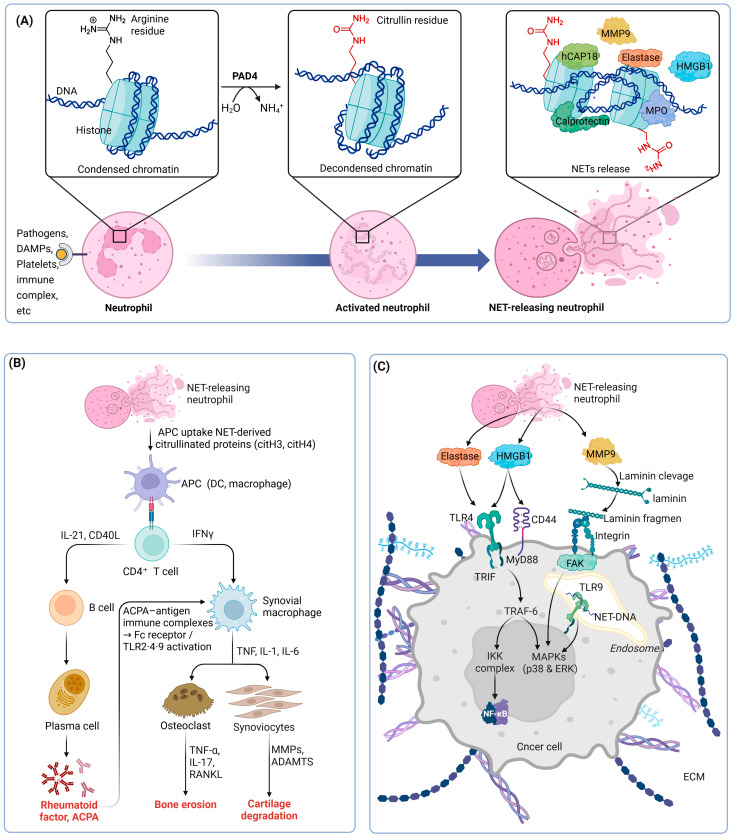
PAD4-Mediated Histone Citrullination Drives NET Formation and Associated Diseases.

Dysregulated PAD4 activity and aberrant NETosis have been increasingly implicated in a broad spectrum of pathological conditions, including autoimmune diseases, cancer, and inflammatory disorders [12,13,14]. In rheumatoid arthritis, NETosis generates PAD4-dependent citrullinated autoantigens that drive anti-citrullinated protein antibody production and sustain chronic synovial inflammation, while NET-derived DNA–histone complexes further amplify inflammatory signaling and promote progressive joint destruction [15]. In cancer, NETosis contributes predominantly to disease progression rather than tumor initiation by capturing circulating tumor cells, promoting immune evasion, and enhancing cancer-associated thrombosis, collectively facilitating metastatic dissemination and adverse clinical outcomes [14,16]. Similarly, in systemic lupus erythematosus, excessive NET formation combined with defective clearance leads to the accumulation of extracellular nuclear antigens, which fuel immune complex formation and persistent type I interferon signaling, thereby establishing a self-amplifying cycle of chronic inflammation and multi-organ tissue damage [14,16]. The mechanisms by which PAD4-mediated histone citrullination promotes NET formation and contributes to disease pathogenesis are illustrated in Figure 1.

Given its central role in disease-associated citrullination and NETosis, PAD4 has emerged as an attractive therapeutic target. Numerous synthetic PAD4 inhibitors have been developed, including irreversible pan-PAD inhibitors such as Cl-amidine and more selective small-molecule inhibitors such as GSK484 and JBI-589 [17,18]. These compounds have demonstrated efficacy in preclinical models of autoimmune disease, inflammation, and cancer by suppressing histone citrullination and NET formation. However, most reported PAD4 inhibitors remain at the preclinical stage, and challenges including limited isoform selectivity, suboptimal pharmacokinetic properties, and potential off-target effects have hindered their clinical translation [17,18]. These limitations highlight the need for alternative strategies that modulate PAD4 activity or PAD4-dependent biological processes without relying exclusively on direct catalytic-site inhibition.

In parallel with synthetic inhibitor development, increasing attention has been directed toward natural products as modulators of PAD4-related pathological processes. While many natural products lack the structural features required for highly potent and selective direct inhibition of the PAD4 catalytic site, multiple studies have reported that natural compounds can influence PAD4-driven pathways indirectly by regulating oxidative stress, calcium signaling, inflammatory transcriptional programs, or NET formation [12,13,14]. These observations suggest that natural products may exert therapeutic effects by modulating PAD4-associated biological processes rather than acting as classical enzyme inhibitors. Accordingly, natural products engage PAD4 through diverse binding modes and regulatory mechanisms, ranging from direct catalytic-site interaction to active-site–adjacent, allosteric, hybrid, and functionally validated modes of modulation. Understanding the mechanisms by which natural products influence PAD4 activity and PAD4-dependent pathways may therefore provide complementary strategies for targeting PAD4-related diseases.

Figure 1A PAD4-mediated histone citrullination and NET formation. In resting neutrophils, chromatin is maintained in a condensed state through electrostatic interactions between positively charged arginine residues on histones and negatively charged DNA. Upon inflammatory stimulation by pathogens, danger-associated molecular patterns (DAMPs), activated platelets, or immune complexes, calcium influx activates peptidyl arginine deiminase 4 (PAD4). PAD4 catalyzes the deimination of arginine residues to citrulline, releasing ammonia and reducing histone positive charge, which leads to chromatin decondensation. Progressive chromatin relaxation facilitates nuclear envelope breakdown and the release of decondensed chromatin decorated with granular and cytoplasmic proteins, including neutrophil elastase, myeloperoxidase (MPO), calprotectin, and hCAP18, into the extracellular space, resulting in the formation of neutrophil extracellular traps. Figure 1B Role of NET-derived citrullinated antigens in rheumatoid arthritis pathogenesis. NET formation releases citrullinated histones (citH3 and citH4), which are taken up by antigen-presenting cells (APCs), including dendritic cells and macrophages. Antigen presentation activates CD4^+^ T cells, which provide help to B cells through IL-21 and CD40–CD40L interactions, promoting plasma cell differentiation and the production of rheumatoid factor and anti-citrullinated protein antibodies (ACPA). ACPA–antigen immune complexes subsequently activate synovial macrophages via Fcγ receptors and Toll-like receptors (TLR2, TLR4, and TLR9), leading to the release of pro-inflammatory cytokines such as TNF-α, IL-1β, and IL-6. These inflammatory mediators, together with IL-17 and RANKL derived from activated T cells and fibroblast-like synoviocytes, promote osteoclast differentiation and bone erosion, while activated synoviocytes produce matrix-degrading enzymes (MMPs and ADAMTS) that drive cartilage degradation. Figure 1C Contribution of NET components to tumor progression and signaling pathways. NET–releasing neutrophils liberate multiple bioactive components, including HMGB1, neutrophil elastase, NET-derived DNA, and matrix metalloproteinase-9 (MMP-9), into the tumor microenvironment. HMGB1 engages cell-surface receptors such as TLR4 and CD44, triggering MyD88-dependent signaling cascades that converge on TRAF6 and activate the IKK complex and downstream NF-κB signaling. NET-derived DNA is internalized into endosomes, where it activates TLR9, leading to MAPK activation, including p38 and ERK. In parallel, NET-associated MMP-9 cleaves laminin, a key component of the extracellular matrix, generating bioactive laminin fragments that activate integrin–FAK signaling in cancer cells. Neutrophil elastase further potentiates TLR4-dependent signaling indirectly through protease-mediated modulation of the extracellular milieu. Together, these converging pathways promote pro-inflammatory, pro-survival, and pro-invasive signaling programs that facilitate tumor progression, extracellular matrix remodeling, and metastatic potential. The cartoon in Figure 1 was created with BioRender.com (https://app.biorender.com, accessed on 2 January 2026).

## 2. Structural Basis of PAD4 and Its Implications for Inhibitor Development

### 2.1. Structure of the PAD4 Active Site

**Figure 2:**
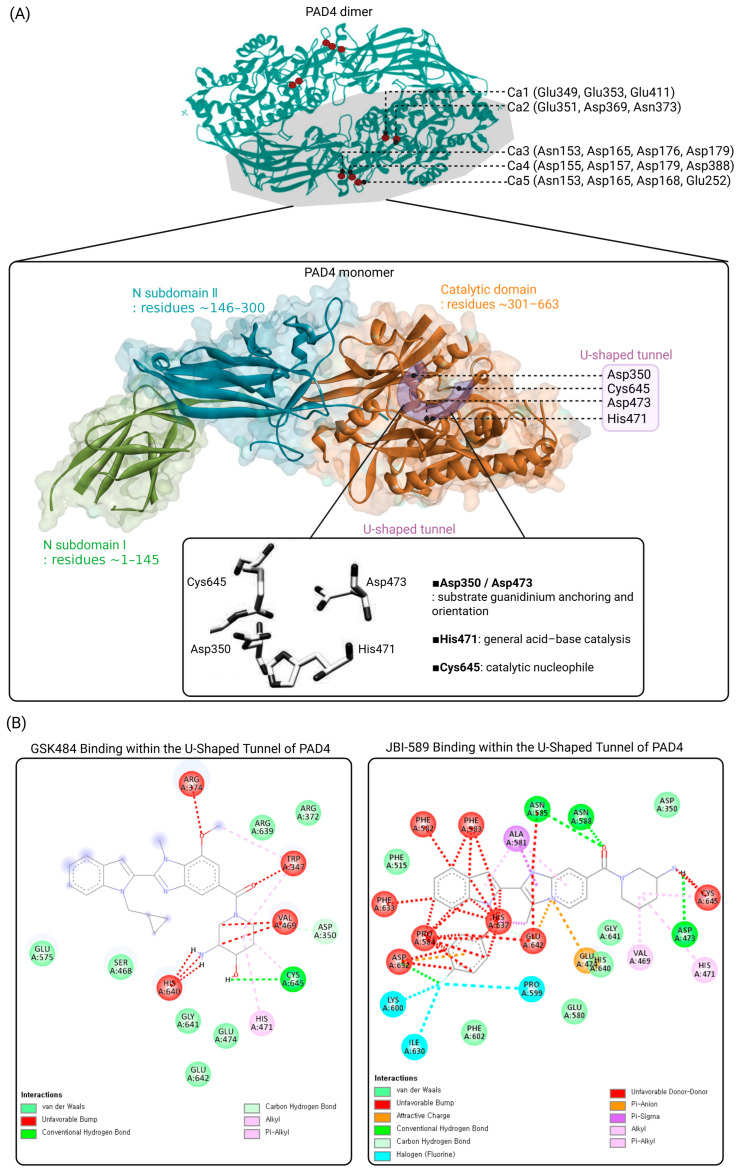
Structural architecture of PAD4 and docking-based binding modes of representative synthetic inhibitors.

PAD4 is a calcium-dependent enzyme that catalyzes the conversion of peptidyl arginine residues into citrulline [12,19]. High-resolution structural studies have demonstrated that PAD4 consists of an N-terminal regulatory domain and a C-terminal catalytic domain, with the active site located within the C-terminal region [19]. In the absence of calcium ions, PAD4 adopts an inactive conformation, whereas calcium binding induces pronounced conformational rearrangements that are required for enzymatic activity [19]. Structural analyses have identified five distinct Ca^2+^-binding sites in PAD4, and occupancy of these sites stabilizes the active conformation of the enzyme by reorganizing the active-site geometry and bringing key catalytic residues into a productive configuration [19,20], as illustrated in Figure 2.

(Figure 2A) Structural organization of PAD4 and catalytic site architecture.

Overall structure of the PAD4 dimer based on the crystal structure (PDB: 1WD9), highlighting calcium-binding sites and domain organization, together with the monomeric structure (PDB: 3B1U) used to illustrate detailed domain architecture and the catalytic site. The N-terminal subdomain I (green), N-terminal subdomain II (cyan), and the C-terminal catalytic domain (orange) are shown in cartoon representation with semi-transparent surface overlay. Five Ca^2+^-binding sites (Ca1–Ca5) are indicated, with representative coordinating residues: Ca1 (Glu349, Glu353, Glu411), Ca2 (Glu351, Asp369, Asn373), Ca3 (Asn153, Asp165, Asp176, Asp179), Ca4 (Asp155, Asp157, Asp179, Asp388), and Ca5 (Asn153, Asp165, Asp168, Glu252). The U-shaped tunnel accommodating arginine-containing substrates is highlighted, and the key catalytic residues Asp350, His471, Asp473, and Cys645 are indicated. A close-up view depicts the spatial arrangement of these residues forming the canonical catalytic core within the tunnel. 

(Figure 2B) Docking analysis of representative synthetic PAD4 inhibitors.

Two-dimensional interaction diagrams showing the predicted binding modes of representative synthetic PAD4 inhibitors docked using CB-Dock2 (https://cadd.labshare.cn/cb-dock2/, accessed on 2 January 2026). The docking simulations were performed using the CB-Dock2 platform, which integrates cavity detection and AutoDock Vina. The crystal structure of human PAD4 (PDB ID: 3B1U) was used as the receptor after removal of water molecules and addition of polar hydrogens. Default docking parameters were applied, and the top-ranked binding poses were selected based on Vina scores and cavity size. Interaction diagrams were generated using Discovery Studio 2024 Client. Left panel: docking pose of GSK484 within the U-shaped active-site tunnel of PAD4, illustrating key hydrogen bonding and hydrophobic interactions with residues lining the catalytic pocket, including proximity to Cys645 and Asp473. Right panel: docking pose of JBI-589 within the same U-shaped tunnel, highlighting extensive hydrophobic packing and polar interactions with residues in the β-hairpin and surrounding catalytic regions. These docking models illustrate how structurally distinct synthetic inhibitors engage the PAD4 active-site tunnel through non-covalent interactions, supporting a structure-based framework for PAD4 inhibitor design.

These Ca^2+^-binding sites are distributed across both the N-terminal and C-terminal domains, where they coordinate large-scale conformational rearrangements required for enzyme activation. Specifically, two Ca^2+^ ions (Ca1 and Ca2) are located in the C-terminal domain near the active-site cleft and are critical for calcium-induced conformational changes, whereas three Ca^2+^ ions (Ca3–Ca5) are positioned in the N-terminal domain and contribute to structural stabilization and proper alignment of catalytic elements. Calcium binding promotes repositioning of key catalytic residues, including Cys645, enabling efficient nucleophilic attack during catalysis. The catalytic core of PAD4 is organized around a negatively charged, U-shaped substrate tunnel that accommodates the arginine side chain and directs it toward the active site, as depicted in Figure 2 [12,17]. This tunnel is formed within the C-terminal catalytic domain and terminates at the catalytic nucleophile Cys645, which lies at the base of the active-site pocket [19,20]. The curved geometry and electrostatic properties of this tunnel enable selective recognition of the positively charged guanidinium group of peptidyl arginine residues, thereby contributing to substrate specificity [12,17].

In addition, PAD4 exists as a head-to-tail homodimer under physiological conditions, and this dimerization is required for full enzymatic activity. Dimer formation is stabilized by hydrophobic interactions and salt bridges between adjacent monomers and is functionally linked to cooperative calcium binding, indicating that Ca^2+^ coordination and dimerization jointly regulate PAD4 activation and catalytic efficiency.

Despite the essential role of Ca^2+^ binding in PAD4 activation, most currently reported PAD4 inhibitors, including both synthetic compounds and natural products, primarily target the catalytic active site or substrate-binding regions. To date, there is a lack of experimental or computational studies specifically designed to directly target the Ca^2+^-binding sites. Given that calcium coordination is indispensable for conformational activation and enzymatic function, the Ca^2+^-binding sites may represent an underexplored regulatory interface and a potential target for future therapeutic development.

Within this region, a limited set of conserved residues defines the functional architecture of the U-shaped tunnel. Asp350, His471, Asp473, and Cys645 are deeply embedded in the active site and constitute the core catalytic machinery of PAD4 [12,17]. Cys645 serves as the nucleophile that attacks the guanidinium carbon during deimination, while His471 functions as a general acid–base catalyst [17,20]. Asp350 stabilizes the protonation state of His471, and together with Asp473, forms a conserved catalytic aspartate pair that anchors and orients the guanidinium group through electrostatic interactions and hydrogen bonding. Importantly, this aspartate pair primarily governs substrate positioning and transition-state stabilization rather than directly participating in nucleophilic chemistry [17]. Collectively, the U-shaped substrate tunnel of PAD4 represents a structurally and electrostatically optimized conduit that links Ca^2+^-dependent conformational activation to precise arginine recognition and efficient catalysis.

### 2.2. Arginine/Guanidinium Specificity and the U-Shaped Substrate Tunnel

PAD4 exhibits strict specificity for peptidyl arginine, which is determined by the three-dimensional architecture of its active site [12,17,20]. Biochemical and structural studies have shown that PAD4 does not efficiently recognize free L-arginine, but instead selectively modifies arginine residues embedded within protein substrates [17,21].

The active site of PAD4 forms a narrow U-shaped substrate tunnel that accommodates the arginine side chain together with portions of the adjacent peptide backbone [17,19]. Within this tunnel, the guanidinium group of arginine is stabilized by salt bridges and hydrogen bonds involving Asp350 and Asp473, while additional interactions with backbone atoms of neighboring residues restrict substrate orientation [12,17,21]. These backbone interactions explain why PAD4 selectively targets peptidyl arginine rather than free amino acids.

This high degree of structural specificity is directly linked to the biological functions of PAD4. PAD4-mediated citrullination of histones H3 and H4 leads to chromatin decondensation by neutralizing the positive charge of arginine residues [5]. This biochemical activity underlies PAD4’s role in transcriptional regulation and in NET formation [7,11]. Genetic or pharmacological disruption of PAD4 activity abolishes histone citrullination and impairs NET formation, demonstrating that precise arginine recognition at the structural level is required for PAD4-driven cellular processes [7,8,11]. 

### 2.3. Structure-Based Development of Representative Synthetic PAD4 Inhibitors

Structural elucidation of PAD4, particularly the identification of a negatively charged U-shaped active-site tunnel harboring the catalytic residue Cys645, has enabled the rational development of synthetic PAD4 inhibitors, which are broadly categorized as irreversible covalent or reversible non-covalent inhibitors [1,18,22]. Early covalent inhibitors, including Cl-amidine and F-amidine, were derived from benzoyl-L-arginine amide–based substrate-mimetic scaffolds and incorporate electrophilic haloacetamidine warheads that irreversibly modify Cys645, exhibiting low micromolar inhibitory potency (IC_50_ ≈ 1–10 μM) and effective suppression of histone citrullination and NET formation in preclinical models [23,24,25,26]. However, their limited isozyme selectivity and off-target reactivity have hindered clinical translation. A major advance was achieved through structure-guided optimization by GlaxoSmithKline, leading to the discovery of GSK199, a highly selective reversible PAD4 inhibitor with submicromolar potency (IC_50_ ≈ 200–300 nM) that binds within the U-shaped active-site tunnel and stabilizes a PAD4-specific β-hairpin region, thereby conferring isozyme selectivity [27]. Further optimization yielded GSK484, which displays enhanced potency (IC_50_ ≈ 100 nM) and preferential binding to the calcium-free conformation of PAD4, highlighting the importance of calcium-dependent conformational dynamics in inhibitor recognition [28]. Building on these scaffolds, advanced heteroaryl-based inhibitors such as JBI-589 were developed, achieving nanomolar potency (IC_50_ ≈ 5–20 nM), excellent selectivity over PAD1–3, and oral bioavailability, with demonstrated efficacy in multiple preclinical models of NET-associated inflammatory diseases. To visualize the binding modes of representative inhibitors, molecular docking analyses of GSK484 and JBI-589 were performed, as detailed binding poses for these inhibitors have not been reported in previous studies. The docking analyses were conducted using the CB-Dock2 platform (https://cadd.labshare.cn/cb-dock2/, accessed on 2 January 2026), which integrates cavity detection and AutoDock Vina-based docking. The docking was performed using the crystal structure of PAD4 (PDB: 3B1U), and the resulting binding poses were selected based on predicted binding affinity and cavity matching. Two-dimensional interaction diagrams were subsequently generated using Discovery Studio 2024 Client to illustrate residue-level interactions within the U-shaped active-site tunnel (Figure 2B). Despite these advances, all reported synthetic PAD4 inhibitors remain at the preclinical or early translational stage, and no compound has yet progressed to late-stage clinical trials.

## 3. Binding Mode-Based Classification of PAD4 Inhibitors from Natural Products

**Figure 3:**
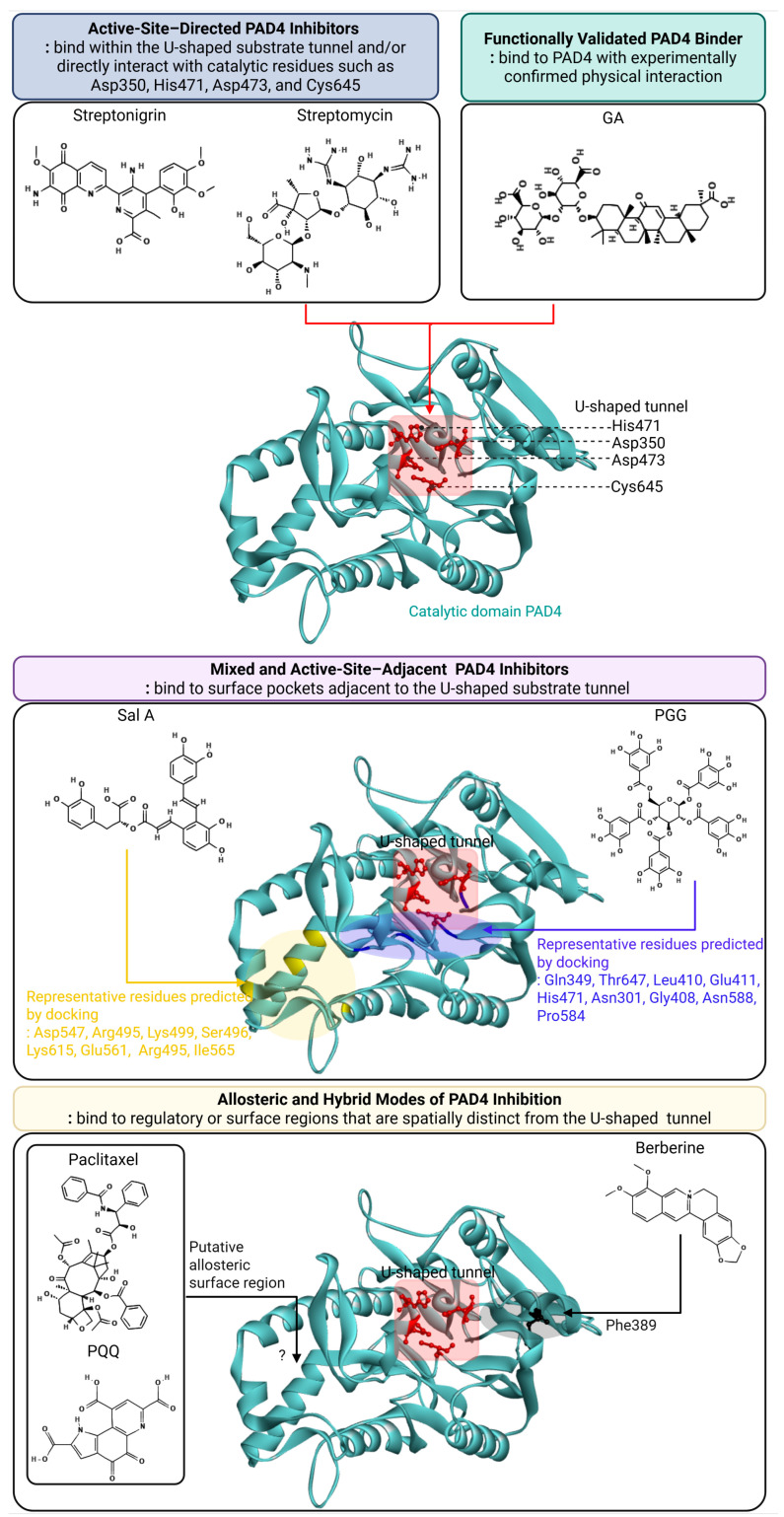
Structural classification of natural product–derived PAD4 inhibitors according to their binding modes and mechanisms of action.

PAD4 modulators can be classified along three independent dimensions: (i) binding mode (active-site–directed, active-site–adjacent, and allosteric), (ii) validation level (experimentally validated vs. putative), and (iii) mechanism of action (direct binding vs. indirect regulation through cellular pathways). These classification axes are considered independently throughout this review. The development of PAD4 inhibitors has historically centered on synthetic small molecules, most notably irreversible haloacetamidine-based compounds such as Cl-amidine and BB-Cl-amidine. These agents were instrumental in establishing the pathological significance of PAD4-driven citrullination and NETosis but remain limited by intrinsic drawbacks, including suboptimal selectivity, metabolic instability, and covalent off-target reactivity. In contrast, recent systematic screening of natural product libraries has uncovered a diverse set of PAD4 modulators that engage the enzyme through multiple binding modes, ranging from direct active-site inhibition to mixed, allosteric, and functionally validated interactions supported by biophysical and cellular evidence, as summarized in Figure 3.

### 3.1. Active-Site-Directed PAD4 Inhibitors from Natural Products

Active-site-directed PAD4 inhibitors are defined as compounds that bind within the U-shaped substrate tunnel and directly interfere with arginine recognition or catalysis by engaging core catalytic residues, including Asp350, His471, Asp473, and the nucleophilic Cys645. Such inhibitors typically compete with the substrate or covalently modify the catalytic machinery, leading to direct suppression of PAD4 enzymatic activity.

Streptonigrin represents one of the most potent natural PAD4 inhibitors identified to date. Identified through Fluopol–activity-based protein profiling (Fluopol-ABPP), streptonigrin inhibits PAD4 with low-micromolar potency (IC_50_ ≈ 2 μM) and high inactivation efficiency [29]. Competitive displacement of activity-based probes that target the catalytic cysteine strongly implicates binding in close proximity to Cys645, providing direct evidence for active-site engagement. The observed time-dependent inactivation kinetics further support irreversible interaction with the catalytic core. Although streptonigrin’s redox activity and cytotoxicity preclude therapeutic development, it provides a critical proof of concept that natural scaffolds can directly access and inactivate the PAD4 active site.

Streptomycin constitutes a mechanistically informative but weak active-site inhibitor. ABPP-based profiling and enzymatic analyses demonstrated that streptomycin inhibits PAD4 in a competitive manner, consistent with direct competition with the peptidyl arginine substrate [30]. Kinetic analyses indicate that streptomycin inhibits PAD4 only at millimolar concentrations (IC_50_ ≈ 1–2 mM), underscoring its low inhibitory potency despite clear evidence of active-site engagement. This behavior is in line with its guanidinium-rich chemical features, which enable substrate-mimetic interaction within the arginine-binding pocket of the catalytic tunnel. Despite its low inhibitory strength, streptomycin provided early experimental evidence that non-amidine natural products can directly engage the PAD4 active site, thereby establishing the enzyme as chemically tractable, as summarized in Table 1.

### 3.2. Mixed and Active-Site-Adjacent PAD4 Inhibitors from Polyphenolic Scaffolds

Mixed and active-site-adjacent PAD4 inhibitors are defined as reversible modulators that bind to surface regions flanking the U-shaped substrate tunnel, rather than directly occupying the arginine-binding pocket, and suppress enzymatic activity through indirect interference with substrate recognition or catalytic efficiency. These inhibitors typically exhibit mixed inhibition kinetics, reflecting their ability to interact with both the free enzyme and the enzyme–substrate complex without classical substrate competition.

Chlortetracycline (CTC) represents an early example of a mixed and active-site–adjacent PAD4 inhibitor identified from natural product–derived scaffolds. Enzymatic analyses revealed that CTC inhibits PAD4 in a reversible mixed manner, with distinct Kis and Kii values, indicating that it does not act through exclusive competition with the substrate [30]. Although its inhibitory potency is relatively weak and confined to the millimolar range, the mixed kinetic profile and lack of evidence for direct catalytic cysteine engagement support an active-site–adjacent binding mode rather than classical active-site blockade. These features place CTC within the mixed, non–substrate-mimetic class of PAD4 inhibitors.

Tetracycline further extends this class of mixed and active-site–adjacent PAD4 inhibitors. Enzymatic studies demonstrated that tetracycline inhibits PAD4 in a reversible mixed manner, with inhibition observed in the high-micromolar to millimolar range (IC_50_ ≈ 0.7–1.0 mM, depending on assay conditions) [30]. Lineweaver–Burk analyses revealed concomitant changes in both Km and Vmax, indicating that tetracycline does not act through classical competitive inhibition. Similar to CTC, tetracycline lacks evidence for direct engagement of the catalytic cysteine Cys645 or covalent modification of the active site, supporting an indirect mode of inhibition. Given its bulky polycyclic scaffold and limited charge complementarity, tetracycline is unlikely to deeply penetrate the narrow U-shaped substrate tunnel and instead is best explained by binding to regions adjacent to the catalytic pocket, where it perturbs substrate access or orientation. These properties place tetracycline within the same mixed, active-site–adjacent inhibitor category as CTC, reinforcing the notion that tetracycline-class antibiotics modulate PAD4 activity through non–substrate-mimetic mechanisms rather than direct active-site blockade.

Pentagalloylglucose (PGG), isolated from Moutan Cortex, exemplifies a reversible PAD4 inhibitor with robust evidence for direct engagement. PGG inhibits PAD4 with low-micromolar potency (IC_50_ ≈ 4–5 μM), and direct binding has been validated by drug affinity responsive target stability (DARTS) assays [31]. Enzyme kinetic analyses indicate mixed inhibition, demonstrating that PGG does not compete exclusively with the substrate for the catalytic pocket. Consistent with this behavior, molecular docking places PGG adjacent to the catalytic region, where it forms extensive hydrogen-bond networks with residues surrounding Cys645 without deeply occupying the narrow U-shaped substrate tunnel, as detailed in Table 2. These findings indicate that PGG modulates PAD4 activity by stabilizing inactive conformations through active-site–adjacent interactions rather than classical active-site blockade, illustrating how bulky polyphenolic scaffolds can achieve reversible inhibition without covalent chemistry.

Salvianolic acid A (Sal A) from Salvia miltiorrhiza further supports this binding paradigm. Identified through bioassay-guided screening, Salvianolic acid A has been identified as a PAD4 inhibitor with an IC_50_ of 33.52 μM, showing mixed inhibition kinetics in enzyme assays [32]. Docking analyses similarly suggest binding to an active-site–adjacent surface region, rather than penetration into the substrate-binding tunnel. This binding mode provides a mechanistic explanation for its mixed kinetic behavior and highlights that PAD4 enzymatic activity can be regulated through noncanonical interactions proximal to, but distinct from, the core catalytic pocket.

### 3.3. Allosteric and Hybrid Modes of PAD4 Inhibition

Allosteric and hybrid PAD4 inhibitors are defined as modulators that suppress enzymatic activity by binding to sites spatially distinct from the U-shaped substrate tunnel, or by engaging multiple interaction modes that combine indirect enzymatic modulation with additional regulatory mechanisms. Unlike active-site–directed inhibitors, these compounds do not rely on direct substrate mimicry and often exert their effects through global conformational regulation, multi-site binding, or combined enzymatic and transcriptional control.

Paclitaxel (Taxol) provided early experimental evidence that PAD enzymes possess allosteric regulatory sites. Kinetic analyses using bovine brain PAD revealed noncompetitive inhibition, characterized by a reduction in maximal velocity without changes in substrate affinity, a kinetic pattern that is incompatible with direct competition at the catalytic pocket and instead implies binding at a spatially distinct regulatory site [33]. Paclitaxel inhibits PAD activity only at millimolar concentrations (typically in the 1–5 mM range), and although it lacks translational relevance as a PAD4 inhibitor, these data established the fundamental principle that PAD enzymatic activity can be modulated independently of direct active-site occupation, thereby providing an early conceptual framework for allosteric PAD inhibition.

Pyrroloquinoline quinone (PQQ) represents a modern example of a mixed-mode PAD4 inhibitor. Identified through sensitive immunoassay-based screening, PQQ inhibits PAD4 with low-micromolar potency across multiple substrates (IC_50_ values ranging from 1.6 to 3.1 μM, depending on the substrate and assay format) [34]. Enzyme kinetic analyses indicate mixed-type inhibition, demonstrating that PQQ does not act solely through substrate competition. Consistent with this behavior, direct binding to PAD4 has been validated by DARTS assays, while molecular docking reveals nonexclusive interactions with multiple regions of the enzyme rather than a single substrate-mimetic pose. Together, these findings support a binding mode that incorporates both active-site–proximal and allosteric components, expanding the conceptual space of PAD4 inhibitor design beyond classical substrate-mimetic frameworks toward redox-active natural scaffolds.

Berberine exemplifies a hybrid PAD4 modulator. Although its direct enzymatic inhibition is modest (IC_50_ ≈ 40–50 μM), enzyme-based assays confirm measurable suppression of PAD4 catalytic activity, and DARTS analysis together with molecular docking support physical interaction between berberine and PAD4 [35]. Importantly, functional studies demonstrate that berberine primarily suppresses PAD4-dependent inflammatory phenotypes through downregulation of PADI4 expression, with direct enzymatic inhibition contributing only partially to its overall biological effect. This combination of direct binding with dominant transcriptional regulation justifies classification of berberine as a hybrid modulator, underscoring that direct PAD4 engagement and catalytic inhibition are not necessarily correlated in magnitude, yet can cooperate to yield biologically meaningful PAD4 modulation.

### 3.4. Functionally Validated PAD4 Binders with Biophysical Support

Glycyrrhizic acid (GA) occupies a distinct mechanistic niche among PAD4-targeting natural products. Although GA lacks measurable enzymatic IC_50_ or Ki values, the compound suppresses PAD4 enzymatic activity in vitro, and surface plasmon resonance experiments demonstrate direct binding to PAD4 with sub-millimolar affinity (KD ≈ 777 μM), supported by molecular docking to the active-site region [36]. Importantly, GA robustly suppresses PAD4-dependent NET formation in cellular and in vivo models. These observations indicate that moderate-affinity binding, when combined with favorable pharmacokinetics and network-level anti-inflammatory actions, can yield biologically meaningful PAD4 inhibition. 

### 3.5. Structural Constraints and Implications for PAD4 Binding Modes

The PAD4 active site is highly specialized for peptidyl arginine recognition, favoring small, positively charged synthetic inhibitors that engage the catalytic aspartate pair or covalently target Cys645 [12,17,21]. In contrast, many natural products possess bulky, polycyclic scaffolds and limited charge density, restricting access to the narrow substrate tunnel. These physicochemical constraints rationalize why natural products more frequently exhibit mixed or allosteric inhibition rather than classical competitive behavior.

Moreover, PAD4 functions as a Ca^2+^-dependent homodimer, and disruption of dimerization or conformational activation reduces enzymatic activity [12,19]. This structural framework supports the concept that natural products may preferentially modulate PAD4 through active-site–adjacent or allosteric regions, rather than by strict substrate mimicry.

### 3.6. Comparative Perspective and Implications for PAD4 Drug Discovery

Collectively, natural products constitute a mechanistically diverse class of PAD4 inhibitors encompassing active-site–directed inhibitors, mixed and allosteric modulators, and functionally validated binders supported by biophysical and cellular evidence. While generally less potent than optimized synthetic inhibitors, compounds such as streptonigrin, PGG, Sal A, PQQ, GA, streptomycin, and berberine provide valuable structural and mechanistic insights. Their ability to combine direct PAD4 engagement with broader anti-inflammatory actions highlights the potential of natural scaffolds as starting points for next-generation PAD4 modulators, particularly when integrated with structure-based optimization and network pharmacology strategies.

**Table 1:** Direct or functionally validated PAD4 inhibitors from natural products. Enzymatic activities were evaluated using biochemical assays such as BAEE-based colorimetric assays (e.g., COLDER assay) and activity-based protein profiling (ABPP). IC_50_ denotes the concentration required to inhibit 50% of enzymatic activity, whereas Ki represents the equilibrium inhibition constant. In mixed inhibition models, Kis and Kii correspond to inhibitor binding to the free enzyme and the enzyme–substrate complex, respectively. Computational docking was performed using AutoDock Vina–based platforms, which predict ligand binding poses and relative binding affinities within defined or predicted binding pockets.

Compound	Enzyme Assay Methods	Potency (IC_50_ or Ki)	Reversibility and Inhibition Mode	Ref.
▪ **Active-Site–Directed PAD4 Inhibitors**				
Streptonigrin	Fluopol-ABPP high-throughput screening; secondary gel-based ABPP assay; BAEE-based PAD activity assay	IC_50_: 1.87 ± 0.24 μM Ki: 8.5 ± 4.3 μM	Reversibility: Irreversible Inhibition mode: Active-site–directed	[29]
Streptomycin	ABPP-based competitive screening assay using rhodamine-conjugated fluoro-amidine (RFA); confirmation by classical PAD4 activity assay measuring citrulline formation from BAEE; kinetic analysis using Lineweaver–Burk plots	IC_50_: 1800 ± 300 μM Ki: 560 ± 170 μM	Reversibility: Reversible Inhibition mode: Competitive	[30]
▪ **Mixed and Active-Site–Adjacent PAD4 Inhibitors**				
CTC	ABPP-based screening (RFA); BAEE-based PAD4 enzymatic assay; detailed kinetic analysis	IC_50_: 100 ± 10 μM; Ki (mixed): Kii = 110 ± 10 μM; Kis = 540 ± 530 μM	Reversibility: Reversible Inhibition mode: Mixed (competitive + uncompetitive)	[30]
Tetracycline	BAEE-based PAD4 enzymatic activity assay	IC_50_: 780 ± 140 μM	Reversibility: Reversible Inhibition mode: Not determined	[30]
PGG	Colorimetric PAD4 activity assay (COLDER method) using BAEE substrate; inhibition kinetics (Michaelis–Menten, Lineweaver–Burk); DARTS assay for target engagement	IC_50_: 4.50 µM	Reversibility: Reversible Inhibition mode: Mixed (noncompetitive + uncompetitive)	[31]
Sal A	Colorimetric PAD4 activity assay using BAEE substrate; inhibition kinetics (Michaelis–Menten, Lineweaver–Burk); reversible inhibition confirmed by time-dependent assay	IC_50_: 33.52 μM; Ki: 21.48 μM	Reversibility: Reversible Inhibition mode: Mixed	[32]
▪ **Allosteric and Hybrid Modes of PAD4 Inhibition**				
Taxol	PAD activity assay using BAEE substrate (citrulline colorimetric assay); MBP deimination assay detected by anti-citrulline antibody (slot-blot); kinetic analysis using V–S and Lineweaver–Burk plots; radiolabeled [^3^H]-paclitaxel binding assay with gel-filtration to confirm direct binding to PAD	Ki: 4500 μM (low BAEE concentrations) Ki: 10,000 μM (high BAEE concentrations)	Reversibility: Reversible Inhibition mode: Noncompetitive	[33]
PQQ	Trypsin-assisted chemiluminescent immunoassay for PAD4 activity; classical COLDER colorimetric assay using BAEE and L-arginine as substrates; inhibition kinetics (Michaelis–Menten and Lineweaver–Burk analysis); DARTS assay to confirm direct target engagement	IC_50_: 1.6 μM (BAEE, COLDER) IC_50_: 2.8 μM (BAEE, immunoassay) IC_50_: 3.1 μM (L-arginine, COLDER)	Reversibility: Reversible Inhibition mode: Mixed (noncompetitive + uncompetitive)	[34]
Berberine	PAD4 citrullination ELISA assay using recombinant human PAD4 and histone H3 substrate; dose–response analysis performed with GraphPad Prism (version 7.0). Direct target engagement further supported by DARTS (Drug Affinity Responsive Target Stability) assay	IC_50_ = 45.07 μM (95% CI: 44.03–46.12 μM)	Reversibility: Reversible Inhibition mode: Not determined	[35]
▪ **Functionally Validated PAD4 Binders**				
GA	Surface plasmon resonance (SPR) binding assay to assess direct interaction between GA and PAD4; functional validation using NETs-related assays (Cit-H3 immunofluorescence, MPO–DNA ELISA, NET formation analysis); pharmacological comparison with PAD4 inhibitor GSK484	KD (SPR): 777 μM IC_50_/Ki: Not reported	Reversibility: Reversible Inhibition mode: Not determined	[36]

**Table 2:** Molecular docking analysis of natural products targeting PAD4, including docking methods, key interacting residues, and predicted binding scores. The calculated Kd values were estimated from docking-derived binding free energies and should be interpreted as approximate indicators rather than absolute thermodynamic constants, particularly because different docking programs employ distinct scoring functions.

Compound	Docking Method	Key Interacting Residues	Docking Score	Ref.
▪ **Active-Site–Directed PAD4 Inhibitors**				
Streptonigrin	Not reported			
Streptomycin	Not reported			
▪ **Mixed and Active-Site–Adjacent PAD4 Inhibitors**				
CTC	Not reported			
Tetracycline	Not reported			
PGG	AutoDock Vina 2.0 (PyRx); protein preparation with AutoDockTools 1.5.6; visualization with PyMOL & Discovery Studio 2020	Gln349, Thr647, Leu410, Glu411, His471, Asn301 (H-bonds); Gly408, Asn588, Pro584 (C–H bonds); Leu140 (π-anion); Cys645 (alkyl); Pro599 (π-alkyl)	−9.6 kcal/mol (Kd ≈ 9.0 × 10^−8^ M)	[31]
Sal A	AutoDock Vina (PyRx); PAD4 structure PDB 3APM; ligand from PubChem; visualization with PyMOL and Discovery Studio 2020	Asp547, Arg495, Lys499, Ser496, Lys615, Glu561 (H-bonds); Arg495 (π-cation); Ile565 (π-alkyl)	−6.7 kcal/mol (Kd ≈ 1.2 × 10^−5^ M)	[32]
▪ **Allosteric and Hybrid Modes of PAD4 Inhibition**				
Taxol	Not reported			
PQQ	AutoDock Vina (PyRx); PAD4 crystal structure PDB ID: 3APM; ligand from PubChem; protein preparation by PyMOL and AutoDockTools	Hydrogen-bond interactions reported (specific residue not explicitly enumerated in text)	−7.3 kcal/mol (Kd ≈ 4.5 × 10^−6^ M)	[34]
Berberine	AutoDock Vina 1.1.2; PAD4 structures from PDB (3APM, 1WD9, 1WD8, 3APN); visualization with PyMOL 2.3.2.	Phe389	−7.45 kcal/mol (Kd ≈ 3.5 × 10^−6^ M)	[35]
▪ **Functionally Validated PAD4 Binders**				
GA	AutoDock Vina; PAD4 crystal structure (PDB ID: 1WD8); ligand from PubChem; protein and ligand prepared using AutoDockTools; interaction analysis with PyMOL	Asp350, His471, Asp473, Cys645 (active-site region)	−8.9 kcal/mol (Kd ≈ 3.0 × 10^−7^ M)	[36]

The catalytic domain of human PAD4 is shown in cartoon representation, with the U-shaped substrate tunnel highlighted and key catalytic residues (Asp350, His471, Asp473, and Cys645) indicated in red. Active-site–directed inhibitors (top left), including streptonigrin and streptomycin, bind within the U-shaped substrate tunnel and directly interfere with arginine recognition or catalysis by engaging core catalytic residues. Functionally validated PAD4 binders (top right), exemplified by glycyrrhizic acid (GA), exhibit experimentally confirmed physical interaction with PAD4, as demonstrated by biophysical assays, and suppress PAD4-dependent biological processes without definitive evidence of classical catalytic-site inhibition. Mixed and active-site–adjacent inhibitors (middle), including chlortetracycline (CTC), pentagalloylglucose (PGG), and salvianolic acid A (Sal A), bind to surface pockets flanking the U-shaped substrate tunnel, leading to mixed inhibition kinetics through indirect interference with substrate recognition or catalytic efficiency. Residues shown represent representative docking-predicted interaction sites. Allosteric and hybrid inhibitors (bottom), such as paclitaxel and berberine, bind to regulatory or surface regions spatially distinct from the substrate tunnel, or engage multiple interaction modes, thereby modulating PAD4 activity through indirect enzymatic regulation and/or additional functional control mechanisms. Putative binding regions are inferred from kinetic analyses and molecular docking studies. The question mark indicates putative or unresolved binding regions inferred from computational predictions but not experimentally validated.

## 4. Putative PAD4 Binders from Natural Products Identified by In Silico Approaches

In addition to experimentally validated PAD4 inhibitors derived from natural products, a growing number of studies have applied in silico approaches to identify natural compounds with the potential to bind PAD4. These efforts typically rely on molecular docking, molecular dynamics (MD) simulations, MM-GBSA (Molecular Mechanics Generalized Born Surface Area) binding energy calculations, and network pharmacology, rather than direct biochemical measurements of enzymatic inhibition. Consequently, the compounds discussed in this section are best described as putative PAD4 binders, whose relevance is inferred from predicted binding modes and pathway-level functional associations rather than from confirmed catalytic blockade.

### 4.1. Structural Hotspots of PAD4 Revealed by Docking Analyses

Despite differences in compound origin and computational workflows, in silico studies converge on several common features of PAD4 ligand engagement [37,38,39,40,41]. Predicted natural product binders are consistently localized to regions overlapping with or adjacent to the catalytic cavity, particularly the substrate-recognition groove and Ca^2+^-responsive active-site environment [37,38,39,40,41]. Across studies, docking poses frequently involve residues such as Asp350 and His471, which are implicated in substrate positioning and catalysis [12,17,19,20,21]. In contrast, direct engagement of the catalytic cysteine Cys645 is rarely predicted, supporting a model in which these compounds interact with PAD4 through non-covalent, reversible binding modes [37,38,39,40,41]. Such interactions are expected to modulate substrate accessibility or local active-site geometry rather than induce irreversible enzymatic inactivation.

### 4.2. Network Pharmacology–Guided Identification of Putative PAD4 Binders

Several investigations combining network pharmacology with molecular docking have identified natural products whose predicted PAD4 binding coincides with suppression of NET-associated inflammatory phenotypes. In these studies, compounds such as berbamine and formononetin were prioritized based on network centrality and disease-module enrichment, followed by docking-based evaluation against PAD4 [37,38]. Docking simulations predicted stable binding within the PAD4 catalytic pocket or substrate-binding groove, with interaction patterns involving polar and aromatic residues surrounding Asp350 and His471. Although these compounds reduced PAD4-dependent readouts in cellular or animal models, direct enzymatic inhibition of PAD4 was not demonstrated, and their effects were often accompanied by changes in PAD4 expression levels. Accordingly, these compounds are best interpreted as network-informed PAD4-associated ligands, rather than confirmed enzymatic inhibitors.

### 4.3. Structure-Based Virtual Screening of Natural Product Libraries

A complementary strategy has employed structure-based virtual screening of natural product libraries against PAD4 crystal structures. Using validated docking protocols, MM-GBSA free-energy calculations, and extended MD simulations, several phytochemicals were predicted to bind PAD4 with high affinity. MD simulations further supported stable ligand occupancy over 100 ns, with hydrogen bonding interactions involving residues such as His471, Asp350, and other residues lining the catalytic channel, although the interacting amino acids vary depending on the ligand [39]. While these results suggest structurally plausible PAD4 engagement, no biochemical or cellular validation was performed, and these compounds remain theoretical binders pending experimental confirmation.

### 4.4. Multi-Target Natural Products with Predicted PAD4 Engagement

Some natural products repeatedly emerge as PAD4-interacting ligands in docking studies while simultaneously targeting other components of the NETosis machinery.

Curcumin exemplifies this category. Docking simulations consistently position curcumin within or near the PAD4 active site, with predicted interactions involving His471, Asp473, and adjacent polar residues [40]. However, the predicted binding poses are shallow and flexible, and curcumin’s well-documented redox activity and transcriptional effects complicate attribution of its biological effects to direct PAD4 engagement. Experimental studies demonstrate suppression of NET formation and reduced PAD4 expression, but quantitative evidence of PAD4 catalytic inhibition is lacking, supporting classification of curcumin as a multi-target NET modulator with putative PAD4 binding. Phenethyl isothiocyanate (PEITC), a dietary isothiocyanate abundant in cruciferous vegetables, has also been proposed as a putative PAD4 binder based on structure-based molecular docking [41]. Docking analysis predicted that PEITC occupies an active-site–proximal pocket of PAD4 with a moderate binding energy (−6.43 kcal/mol), suggesting non-covalent engagement of residues surrounding the catalytic cavity rather than direct interaction with the catalytic cysteine Cys645. Consistent with this prediction, cellular thermal shift assays indicated altered thermal stability of PAD4 upon PEITC treatment, providing supportive but indirect evidence of target engagement. However, residue-level validation and quantitative enzymatic inhibition kinetics were not established, supporting classification of PEITC as a putative PAD4 binder rather than a confirmed catalytic inhibitor.

### 4.5. Distinction from Experimentally Validated Direct PAD4 Inhibitors

The natural products discussed in this section differ fundamentally from the direct PAD4 inhibitors described in Section 3. In contrast to experimentally validated inhibitors, putative PAD4 binders identified through in silico approaches rely primarily on predicted binding poses and energetic favorability, rather than on enzymatic inhibition kinetics or direct measurements of catalytic activity. These compounds are generally predicted to interact with PAD4 through non-covalent binding modes, engaging catalytically relevant regions of the enzyme without inducing irreversible inactivation. Consistent with this model, docking analyses recurrently implicate residues such as Asp350 and His471, whereas direct engagement of the catalytic cysteine Cys645 is rarely observed. Moreover, predicted PAD4 binding is frequently accompanied by indirect regulatory effects, including transcriptional modulation of PADI4 expression and broader multi-target anti-inflammatory activity. Collectively, these features warrant a conservative interpretation of these compounds as putative PAD4 binders rather than confirmed enzymatic inhibitors, while simultaneously highlighting PAD4 as a chemically accessible and structurally permissive target for diverse natural scaffolds.

### 4.6. Outlook

In silico identification of putative PAD4 binders from natural products provides a valuable exploratory framework for expanding the PAD4-related chemical space. However, translation of these predictions into bona fide PAD4 inhibitors requires rigorous experimental validation, including enzymatic inhibition assays, activity-based probe competition, and biophysical binding measurements. When interpreted with appropriate caution, computational studies serve as an essential bridge between systems pharmacology and mechanism-driven drug discovery, guiding rational prioritization of natural products for experimental evaluation. Putative PAD4 binders identified by in silico analysis are presented in Table 3.

## 5. Indirect PAD4 Modulators from Natural Products

### 5.1. Molecular Signaling Pathways Regulating PAD4 Expression and Activation

**Figure 4:**
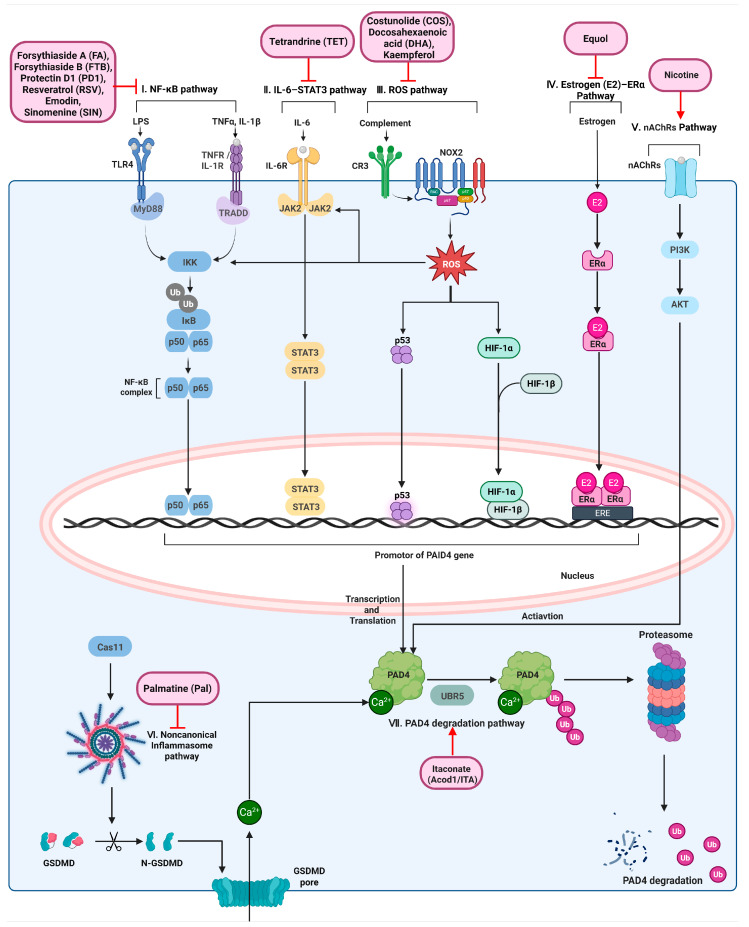
Indirect regulation of PAD4 expression, activation, and degradation by natural products through multiple signaling pathways.

Natural products modulate cellular PAD4 activity primarily through indirect mechanisms that affect upstream signaling pathways rather than by directly inhibiting its catalytic activity. These regulatory pathways operate at multiple levels, including inflammatory transcriptional programs, cytokine-driven signaling cascades, oxidative stress–associated mechanisms, receptor-mediated intracellular signaling, inflammasome activation, and protein degradation processes. Depending on the cellular context, these pathways regulate PAD4 either by controlling PADI4 transcription, modulating PAD4 activation, or influencing PAD4 protein stability. Based on experimentally validated evidence, the following subsections classify indirect PAD4 modulators according to their dominant regulatory signaling axes, with emphasis on the initiating stimuli, intracellular signaling logic, and mechanistic links to PAD4-dependent chromatin remodeling and NET formation, as summarized in Table 4 and Figure 4.

### 5.2. NF-κB and Inflammatory Cytokine–Driven Regulation

The Toll-like receptor (TLR) and cytokine-dependent NF-κB signaling pathway constitutes a major regulatory axis linking inflammatory stimulation to increased PAD4 expression. Activation of NF-κB promotes the transcription of pro-inflammatory cytokines, such as TNF-α and IL-1β, which in turn enhance PAD4 expression in neutrophils and other myeloid cells. Experimental models of acute inflammation have demonstrated parallel increases in NF-κB activity and PAD4 expression, supporting a functional connection between inflammatory transcriptional programs and PAD4 induction [42,43].

Several natural compounds indirectly suppress PAD4 expression and PAD4-dependent NET formation by attenuating NF-κB–driven cytokine signaling. Forsythiaside A reduces TNF-α and IL-6 production in DSS-induced colitis and PMA-stimulated neutrophils, thereby limiting cytokine-mediated PAD4 induction [44]. Forsythiaside B similarly decreases PAD4 protein levels and NET formation in CLP-induced sepsis models, concomitant with reduced TNF-α and IL-6 levels [45]. Protectin D1 suppresses PAD4 expression in activated neutrophils in acute pancreatitis models, accompanied by inhibition of pro-inflammatory cytokine production [46].

In hematological and tumor-associated inflammatory contexts, resveratrol reduces PAD4 mRNA expression and suppresses ETosis in LPS-stimulated NB4 cells in parallel with decreased TNF-α production [47]. Emodin attenuates PAD4 and citrullinated histone H3 (citH3) expression in urethane-induced lung carcinogenesis and N2-polarized neutrophils, consistent with suppression of an NF-κB–dominant inflammatory milieu [48]. Sinomenine inhibits both NF-κB and MAPK signaling pathways, leading to reduced PAD4 and citH3 levels and suppression of NET formation through inhibition of neutrophil autophagy, highlighting pathway crosstalk in inflammatory regulation of PAD4 [49].

### 5.3. IL-6–STAT3 Signaling Axis

The IL-6–gp130–JAK–STAT3 pathway represents a relatively direct signaling axis linking cytokine stimulation to PAD4 transcriptional regulation. In gp130F759 knock-in mice with hyperactive IL-6 signaling, IL-6 stimulation increases Padi4 expression in neutrophils both in vitro and in vivo, accompanied by enhanced STAT3 phosphorylation and elevated PAD4 protein levels in inflamed joints during early inflammatory arthritis [50]. These findings support a model in which STAT3 acts as a key mediator coupling IL-6 signaling to PAD4 induction.

Pharmacological interference with upstream IL-6–associated signaling indirectly suppresses PAD4 expression and NET formation. Tetrandrine inhibits ERK phosphorylation in neutrophils in adjuvant-induced arthritis models and LPS- or PMA-stimulated systems, resulting in reduced production of IL-6 and IL-1β [51]. This reduction in pro-inflammatory cytokines is accompanied by decreased PAD4 and citH3 expression and suppression of PAD4-dependent NET formation, indicating effective disruption of the IL-6–STAT3–PAD4 axis.

### 5.4. Oxidative Stress-Associated Regulation

Inflammatory and complement-derived stimuli activate NADPH oxidase–dependent reactive oxygen species (ROS) production in neutrophils through receptors such as ITGAM and TLR2 [52,53,54]. ROS acts as a redox-sensitive signaling mediator that can influence inflammatory transcription factors, including NF-κB and STAT3 [55,56,57]. Although a direct linear signaling cascade linking ROS to PAD4 transcription has not been fully established, oxidative stress–associated activation of these transcriptional programs is implicated in the regulation of PADI4 expression [42,50]. In parallel, p53 directly transactivates the PADI4 gene in response to genotoxic stress [58], and hypoxia-inducible factor (HIF) has been associated with transcriptional responses under hypoxic and redox stress conditions [59].

Consistent with this mechanism, several natural compounds attenuate PAD4 expression and NET formation by suppressing ROS-associated signaling pathways. Costunolide reduces PAD4 protein levels in STZ/high-fat diet–induced diabetic nephropathy and PMA-stimulated neutrophils by inhibiting ROS-dependent signaling linked to ITGAM–NADPH oxidase activation [60]. Docosahexaenoic acid decreases PAD4 expression in amyloid-β–induced Alzheimer’s disease–like neuronal models in parallel with reduced oxidative stress and inflammation [61]. Kaempferol suppresses NADPH oxidase–derived ROS in neutrophils, leading to reduced histone citrullination and NET formation in a breast cancer metastasis model [62].

### 5.5. Estrogen (E2)–ER-Related Signaling

Estrogen receptor–dependent transcriptional regulation contributes to PAD4 expression in hormone-responsive cellular contexts. Promoter analyses have demonstrated that estrogen stimulation enhances PADI4 transcription through coordinated actions of ERα, AP-1, NF-Y, and Sp1/Sp3 [63].

In innate immune cells, modulation of estrogen-related signaling suppresses PAD4 expression and NET formation. Equol, an estrogen-like metabolite derived from soybean isoflavones, reduces PAD4 mRNA expression and nuclear PAD4 protein levels in LPS-stimulated human neutrophils without impairing phagocytic activity, resulting in decreased NET formation [64].

### 5.6. Nicotinic Acetylcholine Receptor–PI3K–Akt Pathway

PAD4 activity can be regulated through receptor-mediated intracellular signaling pathways independent of canonical inflammatory transcriptional programs. Activation of nicotinic acetylcholine receptors (nAChRs) engages downstream PI3K–Akt signaling in neutrophils [65]. Nicotine induces NET formation in a dose-dependent manner through the nAChR–PI3K–Akt axis, requiring Akt activation and PAD4 activity but occurring independently of NADPH oxidase–derived ROS production. These findings identify nicotine as a PAD4 activator and reveal a non-inflammatory mechanism of PAD4-dependent chromatin decondensation.

### 5.7. Inflammasome-Associated PAD4 Regulation

Noncanonical inflammasome signaling links cytosolic danger sensing to PAD4 activation during NETosis-like responses. Caspase-11–dependent activation of gasdermin D induces calcium influx, which promotes PAD4 activation without directly inducing PADI4 transcription [66].

Natural compounds targeting inflammasome pathways exert distinct regulatory effects on PAD4. Lithocholic acid suppresses PAD4 expression and NET formation by inhibiting canonical NLRP3 inflammasome signaling [67]. In contrast, palmatine reduces PAD4 activation primarily through inhibition of the noncanonical caspase-11–dependent inflammasome axis, resulting in decreased NET formation in inflammatory models [68].

### 5.8. cAMP–PKAc–Dependent Suppression of PAD4

Activation of the neurotensin receptor 1 (NTSR1)–cAMP–PKAc signaling pathway negatively regulates PAD4 expression and histone citrullination. Akebia saponin D enhances NTSR1 expression and activates cAMP/PKAc/CREB signaling, leading to suppression of PAD4 and citH3 expression and inhibition of PAD4-dependent NET formation in both in vivo and in vitro models [69].

### 5.9. Undefined or Incompletely Characterized Mechanisms

Trimethylamine N-oxide suppresses PAD4-dependent NET formation and improves placental and fetal development in gestational diabetes mellitus models; however, the upstream signaling mechanisms responsible for PAD4 modulation remain undefined [70].

**Table 4:** Indirect PAD4 modulators from natural products. The term “highly possible” indicates compounds for which indirect evidence suggests PAD4 inhibition, such as modulation of NET formation, citrullination levels, or PAD4-related signaling pathways, although direct enzymatic or binding assays have not been performed.

Compound	Model	PAD4-Inhibitory Pathway	Major Findings	Ref.
Forsythiaside A (FA)	In vivo: DSS-induced ulcerative colitis in C57BL/6 mice; In vitro: PMA-stimulated neutrophils	NF-κB pathways inhibition (highly possible)	FA reduces TNF-α and IL-6, which are established upstream mediators in NF-κB–linked inflammatory induction of PAD4.	[44]
Forsythiaside B (FTB)	In vivo: CLP-induced sepsis in Sprague–Dawley rats; Ex vivo: PMA-stimulated neutrophils	NF-κB pathways inhibition (highly possible)	FTB reduces PAD4 protein expression and PAD4-dependent NET formation in neutrophils. FTB significantly decreases TNF-α and IL-6 levels, which are established upstream mediators in NF-κB–linked inflammatory induction of PAD4.	[45]
Protectin D1 (PD1)	In vivo: Caerulein-, pancreatic duct ligation–, and L-arginine–induced acute pancreatitis mouse models; In vitro: PMA-stimulated mouse neutrophils	NF-κB pathways inhibition (highly possible)	PD1 reduces PAD4 protein expression in activated neutrophils, accompanied by decreased TNF-α and IL-6 levels, leading to suppression of PAD4-dependent NET formation and attenuation of acute pancreatitis severity.	[46]
Resveratrol (RSV)	In vitro: LPS-stimulated NB4 acute promyelocytic leukemia cells	NF-κB pathways inhibition (highly possible)	RSV decreases PAD4 mRNA expression and suppresses ETOsis, accompanied by reduced TNF-α levels in LPS-stimulated NB4 cells.	[47]
Emodin	In vivo: Urethane-induced lung carcinogenesis in mice; In vitro: N2-polarized neutrophils (HL-60N2)	NF-κB pathways inhibition (highly possible)	Reduces PAD4 and citH3 expression and suppresses NET formation.	[48]
Sinomenine (SIN)	In vivo: adjuvant-induced arthritis (AA) in C57BL/6 mice; In vitro: mouse neutrophils stimulated with LPS or PMA	NF-κB/MAPK pathway inhibition;	SIN reduces p-p65, p-ERK, and p-p38, decreases PAD4 and CitH3 levels, and suppresses NET formation via inhibition of neutrophil autophagy.	[49]
Tetrandrine (TET)	In vivo: Adjuvant-induced arthritis (AA) in C57BL/6 mice; In vitro: LPS- or PMA-stimulated neutrophils	ERK/IL-6 pathways inhibition ↓	Tetrandrine inhibits ERK phosphorylation in neutrophils, leading to reduced production of IL-6 and IL-1β. Suppression of these pro-inflammatory mediators is accompanied by decreased PAD4 and citH3 expression and consequent inhibition of PAD4-dependent NET formation.	[51]
Costunolide (COS)	In vivo: STZ/high-fat diet–induced diabetic nephropathy mouse model; In vitro: PMA-stimulated mouse neutrophils	ROS pathways inhibition	COS reduces PAD4 protein expression by suppressing ROS-dependent signaling linked to ITGAM–NADPH oxidase and by inhibiting inflammatory receptor–associated pathways	[60]
Docosahexaenoic acid (DHA)	In vitro: AA-induced Alzheimer’s disease–like neuronal cell model	ROS pathway inhibition (highly possible)	DHA reduces PAD4 expression, attenuates oxidative stress and inflammation, enhances autophagy-related gene expression, and decreases amyloid-β accumulation, resulting in preserved neuronal integrity.	[61]
Kaempferol	In vivo: 4T1 breast cancer metastasis model (BALB/c mice); In vivo: PAD4^−^/^−^ mice; In vitro: mouse bone marrow–derived neutrophils	ROS pathway inhibition	Kaempferol suppresses NADPH oxidase–derived ROS, leading to reduced PAD4-dependent histone citrullination and NET formation, thereby inhibiting tumor metastasis.	[62]
Equol	In vitro: LPS-stimulated human neutrophils from healthy donors	Estrogen (E2)–ER-related pathway (highly possible)	Equol, an estrogen-like soybean metabolite, reduces PAD4 mRNA expression and nuclear PAD4 protein levels via estrogen-associated signaling, resulting in decreased LPS-induced NET formation in human neutrophils without impairing phagocytic function.	[64]
Nicotine	In vitro: neutrophils (human; plus Nox2-deficient mouse neutrophils used for validation)	Nicotinic acetylcholine receptor–Akt–PAD4 activation	Induces NETs dose-dependently; requires nAChR, Akt, and PAD4; independent of Nox2.	[65]
Lithocholic acid (LCA)	In vivo: DSS-induced acute colitis and Il-10^−^/^−^ chronic colitis mouse models; In vitro: primary murine neutrophils	Canonical NLRP3 inflammasomepathway inhibition	LCA suppresses NET formation and reduces PAD4, MPO, and CitH3 levels in inflamed colon and neutrophils	[67]
Palmatine (Pal)	In vivo: MSU-induced acute gouty arthritis and air pouch models in mice; In vitro: LPS/MSU-stimulated macrophages and neutrophils	Non-canonical inflammasome (caspase-11)–NETs axis inhibition	Reduces caspase-11, PAD4, and histone H3 levels and suppresses NET formation.	[68]
Akebia saponin D (ASD)	In vivo: Intracerebral hemorrhage (ICH) model in rats; In vitro: LPS-stimulated neutrophils	NTSR1–cAMP–PKAc pathway	ASD upregulates NTSR1 and activates the cAMP/PKAc/p-CREB signaling pathway, leading to suppression of PAD4 and citH3 expression and inhibition of PAD4-dependent NET formation in neutrophils.	[69]
Itaconate (Acod1/ITA)	In vivo: CLP- and LPS-induced sepsis models (C57BL/6 mice); In vitro: primary neutrophils	UBR5-mediated ubiquitin–proteasome degradation of PAD4	Acod1-derived itaconate alkylates UBR5, enhances K48-linked ubiquitination of PAD4, and promotes proteasomal PAD4 degradation, leading to reduced NETosis.	[71]
Trimethylamine N-oxide (TMAO)	In vivo: GDM-induced wild-type and PAD4^−^/^−^ mice; In vitro: HTR-8/Svneo trophoblast cells	No experimental evidence	TMAO inhibits PAD4-dependent NET formation and improves placental and fetal development in GDM.	[70]
Curcumin (Cur)	In vivo: ligature-induced periodontitis (LIP) in C57BL/6 mice; In vitro: LPS-stimulated mouse bone marrow–derived neutrophils	No experimental evidence	Curcumin reduces PAD4 protein expression and histone H3 citrullination, directly inhibits LPS-induced NET formation, and alleviates alveolar bone loss in experimental periodontitis.	[40]
Berberine (BBR)	In vitro: U937-derived macrophages; co-culture with A549 lung cancer cells; In vivo: mouse lung carcinogenesis model	No experimental evidence	Berberine downregulates PADI4 expression in macrophages, reverses PADI4-driven pro-tumor macrophage polarization (↓CD163/CD206, ↑IRF5/CD86), and reduces lung tumor nodule formation.	[35]
Formononetin (FMN)	In vivo: DNFB-induced atopic dermatitis in SKH-1 hairless mice and C57BL/6J mice	No experimental evidence	Decreases PAD4, citH3, and MPO expression, leading to suppression of NET formation in lesional skin.	[38]

(I) NF-κB–dependent inflammatory pathway. Inflammatory stimuli such as lipopolysaccharide (LPS) and pro-inflammatory cytokines (TNF-α and IL-1β) activate Toll-like receptor 4 (TLR4) or cytokine receptors, leading to MyD88- and TRADD-dependent activation of the IKK complex, degradation of IκB, and nuclear translocation of the NF-κB p50/p65 complex. NF-κB promotes transcriptional induction of PADI4. Natural compounds including Forsythiaside A, Forsythiaside B, Protectin D1, resveratrol, emodin, and sinomenine suppress PAD4 expression by attenuating NF-κB–driven inflammatory signaling. (II) IL-6–STAT3 signaling axis. IL-6 binding to IL-6 receptor activates JAK2-mediated STAT3 phosphorylation and nuclear translocation, resulting in transcriptional upregulation of PADI4. Tetrandrine inhibits ERK phosphorylation and reduces IL-6 production, thereby indirectly suppressing STAT3 activation and downstream PAD4 expression. (III) ROS-associated pathway. Complement receptor 3 (CR3)–mediated activation of NADPH oxidase (NOX2) leads to intracellular reactive oxygen species (ROS) generation. ROS-associated stress signaling modulates PAD4 expression through redox-sensitive transcriptional regulators, including p53 and hypoxia-inducible factor 1 (HIF-1). Costunolide, docosahexaenoic acid, and kaempferol attenuate PAD4 induction by suppressing ROS-dependent signaling pathways. (IV) Estrogen (E2)–ERα pathway. Estrogen binds estrogen receptor α (ERα), promoting ERα nuclear translocation and binding to estrogen response elements (EREs) within the PADI4 promoter, leading to transcriptional regulation of PAD4. Equol suppresses PAD4 expression through estrogen receptor–associated signaling. (V) Nicotinic acetylcholine receptor (nAChR)–PI3K–Akt pathway. Activation of nAChRs by nicotine engages PI3K–Akt signaling, resulting in PAD4 activation independently of ROS production and inflammatory transcriptional programs, thereby promoting PAD4-dependent chromatin decondensation. (VI) Noncanonical inflammasome pathway. Cytosolic danger signals activate caspase-11–dependent cleavage of gasdermin D (GSDMD), leading to pore formation, Ca^2+^ influx, and subsequent PAD4 activation. Palmatine suppresses PAD4-dependent NET formation by inhibiting caspase-11–mediated noncanonical inflammasome signaling. (VII) PAD4 degradation pathway. PAD4 protein stability is regulated through ubiquitin–proteasome–mediated degradation. Itaconate enhances UBR5-dependent ubiquitination of PAD4, promoting proteasomal degradation and limiting PAD4 availability for NET formation.

## 6. Integrated Perspective and Future Directions for PAD4-Targeting Natural Products

### 6.1. Limitations of Direct PAD4 Inhibition

Despite extensive efforts to develop direct PAD4 inhibitors, translation into clinically effective therapeutics remains limited. Structurally, PAD4 possesses a calcium-dependent catalytic core with a relatively shallow and solvent-exposed active site, which restricts the formation of high-affinity and selective small-molecule inhibitors [28]. The requirement for multiple Ca^2+^ ions to induce the catalytically competent conformation further complicates inhibitor design, as ligand binding and enzymatic activation are tightly coupled to dynamic conformational rearrangements [19].

From a pharmacological perspective, many reported PAD4 inhibitors exhibit suboptimal selectivity across the PAD family or limited bioavailability, raising concerns regarding off-target effects and systemic toxicity [72]. Moreover, complete enzymatic inhibition of PAD4 may not be desirable in all pathological contexts, given its physiological roles in host defense and chromatin regulation [6,11,73,74]. These structural and biological constraints collectively highlight the inherent limitations of strategies that rely solely on direct catalytic inhibition of PAD4.

### 6.2. Indirect Modulation as a Biologically Coherent Strategy

Accumulating evidence indicates that PAD4 functions not merely as an isolated enzyme but as a regulatory node embedded within complex inflammatory, metabolic, and stress-responsive signaling networks. PAD4 activity is governed at multiple levels, including transcriptional regulation, post-translational activation, and protein stability, all of which are dynamically influenced by the cellular microenvironment [2].

Natural products frequently exert their effects upstream of PAD4 by modulating signaling pathways that determine the threshold for PAD4 expression and activation, such as NF-κB–driven inflammatory programs, IL-6–STAT3 signaling, ROS-associated stress responses, and inflammasome activation. Rather than completely abolishing PAD4 function, these compounds fine-tune PAD4-dependent NET formation in a context-dependent manner, thereby reducing pathological NETosis while preserving essential immune functions [13,75].

This mode of action aligns well with the systems-level pharmacology of natural products, which typically target multiple nodes within signaling networks. As such, indirect modulation of PAD4 represents a biologically coherent strategy that reflects the regulatory complexity of PAD4 in vivo.

### 6.3. Direct vs. Indirect PAD4 Targeting: Complementary Rather than Competitive

Direct and indirect PAD4-targeting strategies should not be viewed as mutually exclusive. Direct inhibitors offer the theoretical advantage of high potency and target specificity at the enzymatic level, whereas indirect modulators influence PAD4 activity by reshaping the upstream signaling landscape that governs its expression and activation.

Importantly, indirect modulation may provide disease-context selectivity by preferentially suppressing PAD4 activity under pathological inflammatory conditions while sparing basal PAD4 function in physiological settings. In contrast, direct inhibition may be advantageous in conditions where PAD4 activity is constitutively elevated and tightly linked to disease progression.

From a drug development perspective, combinatorial or sequential strategies integrating direct PAD4 inhibitors with natural product–based indirect modulators may offer synergistic benefits. Such approaches could lower the required dose of direct inhibitors, mitigate adverse effects, and improve therapeutic windows by targeting both the enzymatic activity of PAD4 and its upstream regulatory mechanisms.

### 6.4. Translational Implications and Future Drug Development Opportunities

Biomarker-guided strategies utilizing indicators such as circulating citrullinated histone H3 (CitH3) and cell-free NET-associated DNA have been investigated clinically and show promise for patient stratification and disease severity assessment, suggesting possible applications in guiding PAD4-modulating therapies [76,77,78,79].

Furthermore, disease-specific differences in PAD4 regulation suggest that optimal targeting strategies may vary across pathological contexts, including autoimmune diseases, cancer, infectious diseases, and metabolic disorders. In this regard, natural products provide a valuable source of chemical diversity and biological insight, serving both as lead compounds and as probes to dissect PAD4-regulatory networks [80].

Future PAD4 drug discovery is likely to benefit from integrative approaches combining structural biology, network pharmacology, and immunopathology. Rather than focusing exclusively on catalytic inhibition, targeting the regulatory architecture surrounding PAD4 may yield safer and more effective therapeutic strategies.

## 7. Conclusions

PAD4 has emerged as a central regulator of histone citrullination, chromatin dynamics, and NET formation, linking innate immune activation to diverse pathological outcomes. Structural studies have established PAD4 as a chemically tractable but highly constrained enzymatic target, characterized by calcium-dependent activation and a narrow, negatively charged catalytic tunnel optimized for peptidyl arginine recognition. These features have enabled the development of potent synthetic inhibitors, yet they simultaneously impose limitations on selectivity, druggability, and long-term safety.

Natural products expand the therapeutic landscape of PAD4 modulation by offering mechanistically diverse modes of action that extend beyond direct catalytic inhibition. Although only a small subset of natural compounds directly bind PAD4 with measurable enzymatic inhibition, many exert robust biological effects by regulating PAD4 expression, activation, or degradation through upstream inflammatory, oxidative, hormonal, and inflammasome-associated signaling pathways. Such indirect modulation allows fine-tuning of PAD4-dependent NETosis in a context-dependent manner, potentially preserving physiological host-defense functions while suppressing pathological hyperactivation.

Rather than competing strategies, direct and indirect PAD4 targeting should be viewed as complementary approaches. Direct inhibitors may be advantageous in conditions characterized by sustained PAD4 hyperactivity, whereas indirect modulators may provide disease-context selectivity by reshaping the regulatory environment that governs PAD4 function. Integrative strategies combining structural optimization, network pharmacology, and biomarker-guided patient stratification are likely to define the next phase of PAD4-targeted drug discovery.

Collectively, this review underscores the value of natural products not only as sources of lead compounds but also as biological probes that illuminate the regulatory architecture surrounding PAD4. Targeting PAD4 within its native signaling networks, rather than focusing exclusively on enzymatic blockade, may ultimately yield safer and more effective therapeutic strategies for PAD4-associated diseases.

## Figures and Tables

**Table 3:** Putative PAD4 binders (in silico). The calculated Kd values were estimated from docking-derived binding free energies and should be interpreted as approximate indicators rather than absolute thermodynamic constants, particularly because different docking programs employ distinct scoring functions.

Compound	Origin	In Silico Method	Predicted PAD4 Binding Region/Key Residues	Predicted Binding Energy	Ref.
Berbamine	*Berberis vulgaris* L.	Network pharmacology–guided molecular docking (AutoDock Vina via PyRx)	Catalytic pocket vicinity; Asp350, His471	~−9.3 kcal/mol (Vina score) (Kd ≈ 1.5 × 10^−7^ M)	[37]
Formononetin (FMN)	*Sophora flavescens* Aiton (Kushen)	Network pharmacology–integrated molecular docking (AutoDock Vina via PyRx)	Substrate-recognition groove; no Cys645 engagement	−7.8 kcal/mol (Vina affinity) (Kd ≈ 2.0 × 10^−6^ M)	[38]
Compound: NF_35	*Morinda citrifolia* (noni fruit)	Structure-based virtual screening (Glide XP docking + MM-GBSA + 100 ns MD simulation)	Catalytic channel; His471, Asp350	XP Glide: −8.866 kcal/mol; MM-GBSA ΔG: −60.711 kcal/mol (Kd ≈ 3.2 × 10^−7^ M)	[39]
Curcumin (Cur)	*Curcuma longa* (turmeric)	Network pharmacology–based molecular docking (AutoDock Vina)	Shallow active-site region; His471, Asp473	−6.5 kcal/mol (Vina affinity) (Kd ≈ 1.8 × 10^−5^ M)	[40]
Phenethyl isothiocyanate (PEITC)	*Cruciferous vegetables* (e.g., broccoli, watercress)	Molecular docking (AutoDock-based); CETSA as supportive evidence	Active-site-proximal pocket; (specific catalytic residue engagement not fully validated)	−6.43 kcal/mol (AutoDock score) (Kd ≈ 2.0 × 10^−5^ M)	[41]

## Data Availability

No new data were created or analyzed in this study. Data sharing is not applicable to this article.

## Abbreviations

The following abbreviations are used in this manuscript:

**Abbreviation**	**Full Name**
ABPP	Activity-Based Protein Profiling
ACPA	Anti-Citrullinated Protein Antibody
ADAMTS	A Disintegrin And Metalloproteinase with Thrombospondin Motifs
APC	Antigen-Presenting Cell
B cell	B Lymphocyte
BAEE	Nα-Benzoyl-L-Arginine Ethyl Ester
CD4+ T cell	Cluster of Differentiation 4 Positive T Cell
CD40	Cluster of Differentiation 40
CD40L	CD40 Ligand
CD44	Cluster of Differentiation 44
CETSA	Cellular Thermal Shift Assay
citH3/citH4	Citrullinated Histone H3/Citrullinated Histone H4
CTC	Chlortetracycline
DAMPs	Damage-Associated Molecular Patterns
DARTS	Drug Affinity Responsive Target Stability
DC	Dendritic Cell
DNA	Deoxyribonucleic Acid
ECM	Extracellular Matrix
ERK	Extracellular Signal-Regulated Kinase
FAK	Focal Adhesion Kinase
FcγR	Fc Gamma Receptor
GA	Glycyrrhizic Acid
HMGB1	High Mobility Group Box 1
IC50	Half Maximal Inhibitory Concentration
IFNγ	Interferon Gamma
IKK	IκB Kinase
IL-1β	Interleukin 1 Beta
IL-17	Interleukin 17
IL-21	Interleukin 21
IL-6	Interleukin 6
KD	Dissociation Constant
Ki	Inhibition Constant
MAPK	Mitogen-Activated Protein Kinase
MD	Molecular Dynamics
MMP/MMP-9	Matrix Metalloproteinase/Matrix Metalloproteinase-9
MM-GBSA	Molecular Mechanics Generalized Born Surface Area
MPO	Myeloperoxidase
MyD88	Myeloid Differentiation Primary Response 88
NET-DNA	Neutrophil Extracellular Trap-Derived DNA
NET/NETs	Neutrophil Extracellular Trap(s)
NETosis	Neutrophil Extracellular Trap Formation
NF-κB	Nuclear Factor Kappa-Light-Chain-Enhancer of Activated B Cells
PAD4	Peptidyl Arginine Deiminase 4
p38	p38 Mitogen-Activated Protein Kinase
PGG	Pentagalloylglucose
PQQ	Pyrroloquinoline Quinone
RANKL	Receptor Activator of Nuclear Factor Kappa-B Ligand
RSV	Resveratrol
Sal A	Salvianolic Acid A
SIN	Sinomenine
SPR	Surface Plasmon Resonance
TLR2	Toll-Like Receptor 2
TLR4	Toll-Like Receptor 4
TLR9	Toll-Like Receptor 9
TNF-α	Tumor Necrosis Factor Alpha
TRAF6	TNF Receptor Associated Factor 6
